# Validating feature tracking MRI for the assessment of strain rate in patients with various hemodyanamic states

**DOI:** 10.1186/1532-429X-14-S1-O38

**Published:** 2012-02-01

**Authors:** Dvorah Holtzman, Jeannette McLaughlin, Madhavi Kadiyala, Peter D Rhee, Jie J Cao

**Affiliations:** 1 St. Francis Hospital Roslyn, Long Island, Greenvale, NY, USA

## Background

Myocardial strain rate (SR) is a valuable measurement in assessing myocardial mechanical properties, which can be compared between subjects in the setting of various hemodynamic states. Feature tracking (FT) is a novel method to assess SR off-line on cine images acquired in routine cardiac MRI. In this study we sought to assess SR using the FT method in a group of patients and to validate the relation of SR to LV ejection fraction (LVEF) and left ventricular end diastolic pressure (LVEDP) measured during cardiac catheterization.

## Methods

Fifty two patients undergoing cardiac catheterization and same day research cardiac MRI were prospectively recruited. SR was analyzed in endocardial cine images of the short axis in the mid left ventricle using FT-MRI (Diogenes TomTec Imaging Systems). Average systolic circumferential SR and radial SR were determined. FT strain parameters were compared to the LV ejection fraction (LVEF), LV end systolic (LVESV) and end diastolic volume (LVEDV), and LVEDP obtained during cardiac catheterization.

## Results

Mean age of the cohort was 59 years with 32 patients being male (61%). The average LVEF was 49±15%, LVEDV 90±38 mL/m2 and LVEDP 14±7 mmHg. The average circumferential SR was higher in patients with a normal LVEF (≥50%) -1.6±0.4s-1 versus patients with a low LVEF (<50%) -0.7±0.4s-1 (p<0.001). Significant differences were also noted in average radial SR in patients with normal LVEF 2.1±0.8s-1 versus 0.9±0.5</s-1 (p<0.001) in patients with a low LVEF. While average strain rates were consistently higher in patients with a normal LVEF, there is a lack of linear correlation between circumferential SR and radial SR with LVEF (p=0.241 and 0.987, respectively). In contrast, among patients with low LVEF, the correlation was excellent with r=0.810 (p<0.001) for circumferential SR and r=0.730 (p=0.002) for radial SR. In univariate analysis LVEDP significantly correlated with circumferential SR and radial SR (r=0.579, p<0.001 and r=-0.438, p=0.001, respectively). However, in a multivariate regression model including LVEDP, LVEF, LVEDV, and LVESV as covariables to assess their relationship to circumferential SR and radial SR, LVEDP was not significantly associated with either SR measurements while LVESV and LVEF were significantly associated with circumferential SR and radial SR.

## Conclusions

FT-MRI is a promising method to assess SR in off-line standard cine images. SR assessed by FT is sensitive to change in global LV dysfunction yet independent of LV preload condition.

## Funding

None.

